# Emergence of a virulent porcine reproductive and respiratory syndrome virus in Taiwan in 2018

**DOI:** 10.1111/tbed.13173

**Published:** 2019-04-09

**Authors:** Wei‐Hao Lin, Hsing‐Chun Shih, Sheng‐Yuan Wang, Chuen‐Fu Lin, Cheng‐Yao Yang, Ming‐Tang Chiou, Chao‐Nan Lin

**Affiliations:** ^1^ Department of Veterinary Medicine, College of Veterinary Medicine National Pingtung University of Science and Technology Pingtung Taiwan; ^2^ Animal Disease Diagnostic Center, College of Veterinary Medicine National Pingtung University of Science and Technology Pingtung Taiwan; ^3^ Department of Veterinary Medicine, College of Veterinary Medicine National Chiayi University Chiayi Taiwan; ^4^ Graduate Institute of Veterinary Pathobiology, College of Veterinary Medicine National Chung Hsing University Taichung Taiwan; ^5^ Research Center for Animal Biologics National Pingtung University of Science and Technology Pingtung Taiwan

**Keywords:** emerging, porcine reproductive and respiratory syndrome virus, Taiwan, virulent

## Abstract

In March 2018, an abortion storm caused by porcine reproductive and respiratory syndrome virus was confirmed in a farrow‐to‐finish pig herd in Taiwan. Open reading frame 5 and non‐structural protein 2 of the virus confirmed that the virus is closely related to the virulent strains circulating in the United States.

## 1 INTRODUCTION

Porcine reproductive and respiratory syndrome (PRRS) causes significant economic losses in most pig‐producing countries worldwide (Zimmerman, 2012). The causative agent, PRRS virus (PRRSV), is a single‐stranded, positive‐sense enveloped RNA virus with a genome size of approximately 15 kb, and it belongs to the family *Arteriviridae* and order *Nidovirales*. PRRSVs are divided into European (EU genotype, type 1) and North American (NA genotype, type 2) genotype that vary in the full length of their nucleotide sequences by approximately 60% (Murtaugh, Elam, & Kakach, 1995; Nelsen, Murtaugh, & Faaberg, 1999; Wensvoort et al., 1992). Recently, the emergence of virulent strains of PRRSVs has been reported in the US, such as the NADC30 strain (Brockmeier et al., 2012) or MN414 strain (Wang, Marthaler, Rovira, Rossow, & Murtaugh, 2015). These virulent PRRSV strains have 131 amino acid (aa) discontinuous deletions in non‐structural protein 2 (NSP2), which can be distinguish themselves from other PRRSV strains (Brockmeier et al., 2012; Zhou et al., 2015). Surprisingly, these MN184‐like/NADC30‐like strains of PRRSV are currently emerging on the Asian continent, including China and Korea (Choi et al., 2014; Zhou et al., 2015). Taiwan has been considered free from highly pathogenic PRRSV (HP‐PRRSV) and MN184‐like/NADC30‐like strains of PRRSV based on the latest report of deletion/insertion pattern analysis for the NSP2 gene of PRRSV (Deng et al., 2015). We report the emergence of a virulent PRRSV isolate in a PRRSV‐vaccinated herd in Taiwan, designated PRRSV/TW/NPUST‐107‐844/2018 (NPUST‐107‐844/2018), that had identical deletion patterns to that in virulent PRRSV isolated in the US.

## 2 MATERIALS AND METHODS

### 2.1 Sample collection and history

In March 2018, a 300‐sow farrow‐to‐finish herd with a one‐year PRRSV (Ingelvac PRRSV MLV, Boehringer Ingelheim, St. Joseph, MO) vaccination history (mass vaccinated four times per year) that suffered by an abortion storm (30 abortions within 1 month) was observed in southern Taiwan. The farm had introduced a boar from a domestic breeding herd without quarantine one month prior. Aborted fetuses and whole blood from the corresponding sows were submitted to the Animal Disease Diagnostic Center, National Pingtung University of Science and Technology, Taiwan.

### 2.2 Detection of porcine reproductive and respiratory syndrome virus

Two serum samples from diseased sows and two aborted fetuses from the other diseased sow, including lung, heart, spleen, placenta, and umbilical cord, were mixed and extracted nucleic acid by MagNA Pure LC 2.0 with the MagNA Pure LC Total Nucleic Acid Kit (Roche Applied Science, Indianapolis, IN). Following cDNA synthesis was using PrimeScript™ RT reagent kits (Takara, Kyoto, Japan). All samples were examined by real‐time polymerase chain reaction (Lin, Lin, Hung, Wang, & Chiou, 2013).

### 2.3 Gene amplification and sequencing

The complete open reading frame 5 (ORF5) genes and partial NSP2 genes were amplified as previous studies with some primer modifications for Taiwanese PRRSV, Nsp2‐F3: 5′‐GAR‐GAG‐GTS‐RSR‐RVW‐AAR‐ATT‐G‐3′; Nsp2‐R3: 5′‐CCR‐CCH‐GWG‐TCR‐ATG‐ATG‐GCT‐TG‐3′, respectively (Deng et al., 2015; Zhang et al., 2017). The correct size amplicons were cloned into a TA cloning vector (Yeastern Biotech, Taipei, Taiwan). The plasmids were extracted by Plasmid Midiprep Purification Kit (Genemark, Taipei, Taiwan) and submitted to Mission Biotech Company (Taipei, Taiwan) for sequencing. The sequences were determined from both orientations by an ABI 3730XL DNA analyzer (Applied Biosystems, Foster City, CA).

### 2.4 Sequencing alignment and phylogenic analysis

Sequences of ORF5 gene and NSP2 amino acid were aligned using Clustal W method and Muscle method in Molecular Evolutionary Genetics Analysis Version 6.0 (MEGA6) software, respectively (Tamura, Stecher, Peterson, Filipski, & Kumar, 2013). The sequence homology of nucleotide and amino acid were determined by the MegAlign software (Lasergene, DNASTAR, Madison, WI). Phylogenetic tree was constructed from the aligned nucleotide sequences using the Maximum Likelihood method and the Kimura 2‐parameter model by MEGA6 software (Kimura, 1980). The percent frequencies of the groupings were determined after 1,000 bootstrap evaluation.

## 3 RESULTS

**Figure 1:**
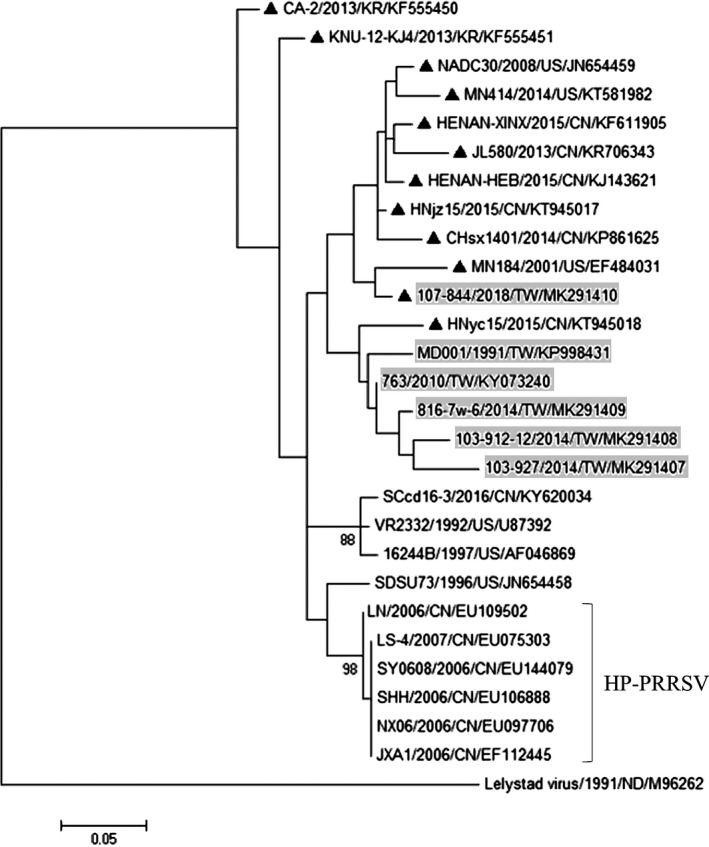
Phylogenetic tree constructed on the basis of the completed open reading frame 5 (ORF5) sequences of PRRS virus (PRRSV). The dendrogram was constructed using the maximum likelihood method based on the Kimura 2‐parameter model in MEGA software, version 6.0. The number of branches was calculated using bootstrapped values from 1,000 replicates. Triangles represent MN184‐like/NADC30‐like strains of PRRSV. Gray underlay represents local PRRSV strains. The European prototype strain Lelystad virus was used as the outgroup

All samples were PRRSV RNA‐positive, and viral loads in the two serum samples were 1.14 × 10^5^ (PRRSV/TW/107‐844/2018) and 3.88 × 10^3^ genomes/μl, respectively. The completed ORF5 sequence of NPUST‐107‐844/2018 was compared to 27 PRRSV sequences obtained from GenBank. The PRRSV ORF5 gene comparative analyses revealed that NPUST‐107‐844 had 88%, 88.8% and 89.8% nucleotide identity (86.3%, 91.7% and 92.2% identity for deduced amino acids) with VR2332, NADC30 and MN184, respectively. NPUST‐107‐844 had lower nucleotide (83.7%–85.9%) and amino acid (84.9%–87.4%) identities with Taiwanese PRRSV. NPUST‐107‐844 had 83%–88.7% and 88%–89.2% nucleotide identity (83.4%–92.7% and 92.2%–92.7% identity for deduced amino acids) with Chinese and Korean MN184‐like/NADC30‐like PRRSVs, respectively. Phylogenetic tree based on the completed ORF5 nucleotide sequence showed that NPUST‐107‐844 was genetically more closely related to MN184 and clustered into a specific branch together with MN184‐like/NADC30‐like strains, MN414/2014, and NADC30 of PRRSVs (Figure 1).

**Figure 2:**
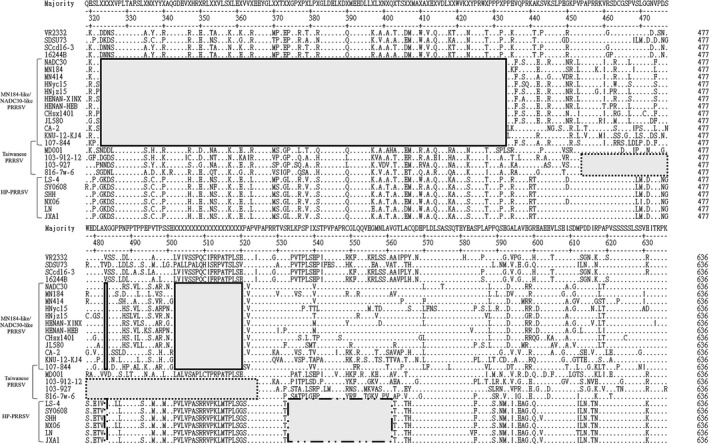
Alignment of amino acid sequences of non‐structural protein 2 (NSP2) from 27 PRRS virus (PRRSV) strains. The deletion pattern of MN184‐like/NADC30‐like, local and highly pathogenic PRRSVs (HP‐PRRSVs) are boxed in solid lines, dotted lines and dash‐dot‐dot lines, respectively

Amino acid alignment of the NSP2 highly variable region of NPUST‐107‐844 displayed 131 aa discontinuous deletions, which were identified as a 111‐aa, 1‐aa and 19‐aa deletion pattern compared with the sequence of the prototype strain VR2332. These deletion patterns were identical to those in NADC30 and MN184 isolates in the US, CA‐2 and KNU‐12‐KJ4 in Korea and MN184‐like/NADC30‐like strains (HENAN‐XINX, CHsx1401, and JL580) in China (Figure 2). Interestingly, there were 231, 0, and 0 breeding pigs imported from US, Korea and China, respectively, between 2015 and 2017. These results confirmed that NPUST‐107‐844 was a novel PRRSV in Taiwan and probably originated in the US. Up to the present, there are no other outbreaks in the domestic breeding herd and other pig farms in southern Taiwan based on our surveillance. Additional PRRSV cases need to be investigated using continuous surveillance and sequence analysis.

In conclusion, this is the first case report of a novel virulent PRRSV in a Taiwanese pig herd, which shares a common evolutionary origin in NSP2 with other virulent PRRSVs in the US, Korea and China. When and how this virus was introduced into Taiwan remains unknown. However, we propose that this virulent strain was introduced into Taiwan in recent years by importing boars and sows from abroad. Further surveillance is worthwhile to define the distribution of this virulent strain of PRRSV among the Taiwanese pig population. These results will also provide the valuable information about the biosecurity concerns (breeding pigs from domestic pig farms as well as from abroad) for the control of PRRSV infection.

### 3.1 Nucleotide sequence accession numbers

The PRRSV sequences obtained in this study have been deposited in GenBank under accession numbers MK291407–MK291414.

## AVAILABILITY OF DATA AND MATERIALS

The data set supporting the conclusions of this article is available in the GenBank.

## CONFLICT OF INTEREST

The authors declared that they have no competing interests.
